# How comprehensive and effective are waste management policies during the COVID-19 pandemic? Perspectives from the Philippines

**DOI:** 10.3389/fpubh.2022.958241

**Published:** 2022-08-17

**Authors:** Geminn Louis Carace Apostol, Angelina Gabrielle Aguilar Acolola, Michelle Alexandra Edillon, Sary Valenzuela

**Affiliations:** Center for Research and Innovation, Ateneo de Manila University–School of Medicine and Public Health, Pasig, Philippines

**Keywords:** policy analysis, COVID-19, waste management, solid waste, healthcare waste, pandemic, Philippines

## Abstract

This study presents a comprehensive analysis on policies governing the management of COVID-19 waste in the Philippines, highlighting gaps in pre-existing policies and opportunities for further policy development and adaptation in the context of present and future public health emergencies. A hybrid search strategy and consultative process identified fifty (50) policy documents directly impacting the management of wastes (general domestic, healthcare, and household healthcare waste) released prior to and during the pandemic. Content analysis revealed comprehensive policy coverage on managing general domestic waste and healthcare waste. However, there remains a dearth in policies for managing household healthcare waste, an emerging category for waste generated by patients isolating at home or in isolation facilities. Applicable, pre-existing policies were neither adequate nor specific to this category, and may therefore be subjected to variable interpretation and mismanagement when applied to this novel waste category. Assessment using the modified Cradle-to-End-Of-Life (CTEOL) framework revealed adequate policy coverage across the waste lifecycle stages. However, policies on reducing waste generation were relatively minimal and outdated, and policy gaps in waste segregation led to downstream inefficiencies and introduction of environmental health risks in waste collection, treatment, and disposal. The internal validity of policies was also evaluated against eleven (11) criteria adapted from Rütten et al. and Cheung et al. The criteria analysis revealed strong fulfillment of ensuring policy accessibility, goal clarity, provision of human resources, and strength of policy background, but weak fulfillment of criteria on providing adequate financing, organizational capacity building, monitoring and evaluation, and encouragement of opportunities for public participation. We conclude that existing waste management policies in the Philippines leave much room for improvement to ensure effective management of COVID-19 waste from various settings and circumstances. Hence, these policies are expected to adapt and evolve over time, utilizing the best available technology and environmental practices. Integrated, region-wide waste management systems, involving both government and society, and strengthened by equitable provisional support are needed for effective waste management that is both inclusive and resilient in the face of present and future pandemics.

## Introduction

The ongoing Coronavirus 2019 (COVID-19) has garnered over 554 million confirmed cases all over the world (1). The Philippines is one of the worst-hit nations globally (2) and in Southeast Asia (3) with more than 3.7 million recorded cases and counting (4). Consequently, the country has also observed a sharp rise in waste generated from both healthcare and domestic settings (5). In April 2020, early into the pandemic, the Asian Development Bank already projected Metro Manila would generate 280 tons of healthcare waste per day, amounting to roughly a 500% increase in solid waste generation from a pre-COVID baseline figure of 52 tons per day (6, 7). In comparison, it was reported that in Wuhan, China, infectious medical solid waste had gone up daily by over 600% from 40 tons daily pre-COVID outbreak, to 270 tons daily during the outbreak (8). More than a year later, the country's Department of Environment and Natural Resources (DENR) reported that the Philippines had in fact generated 634,687.73 metric tons of healthcare waste between June 2020 and June 2021. This means that in a month, the country generates roughly 52,890 MT of healthcare waste alone, exceeding preliminary estimates (9). Specifically, the country is estimated to discard 41,202,485 face masks per day (10). With regards to general solid waste, the Philippines is estimated to generate 8,218,580.85 tons of plastic waste annually as the pandemic progresses (10), which is a dramatic increase from the 2.7 million tons of plastic waste produced in 2019 (11). This surge in “COVID-19 waste” threatens not only the public's health but also the sustainability of the country's already overburdened waste management chains.

In the context of a rapidly progressing pandemic, it is a given that medical waste will inevitably be multiplied as a result of the increased need for commodities and personal protective equipment (PPE) in healthcare settings. A waste audit report released by Health Care Without Harm-Southeast Asia (HCWH) conducted in five major hospitals in Metro Manila confirms this inevitable increase in infectious waste generation, but also highlighted the unnecessary and avoidable increase in the generation of single-use plastic wastes in healthcare facilities (12).

In parallel, the rise in domestic waste during the pandemic, predominantly single-use plastics, was also observed in the Philippines and globally (13). Such rise in domestic waste generation has been largely attributed to public demand for increased health protection, such as the mandatory use of PPEs in public, increased frequencies of home-based health screening and monitoring, and increased frequency of personal and environmental disinfection) (10, 14–16). These have likewise been linked to changes in consumer behavior during the pandemic, specifically, the increase in food takeaways and e-commerce transactions among localities placed on lockdown and imposed with mobility restrictions (17–20).

The exponentiating generation of both healthcare and domestic during the pandemic poses a critical problem for both the public's public health and the environment. Mismanagement of infectious medical waste from healthcare facilities and improper segregation of potentially infectious waste from patients isolating at home may lead to further spread of infection (8). Furthermore, the disruption of the waste management system at the domestic level may lead to open burning and open dumping, with the marine and terrestrial ecosystems bearing the brunt of their environmental effects (21, 22). As cases remain steady in number, with the possibility of subsequent COVID-19 surges, waste management chains soon risk collapse with compounded social, economic, and environmental consequences for the country.

Preserving the integrity of the waste management chain through legislation and policy-making is critical in both containing COVID-19 transmission and mitigating further environmental pollution (23). Even prior to the COVID-19 pandemic, the Philippines already had in place a number of national, subnational, and even local policies that govern the management of both general solid wastes and healthcare wastes. Notably, the country's Ecological Solid Waste Management Act of 2000 (RA 9003) was crafted to ensure the protection of public health and the environment through the utilization of environmentally sound methods for treating, handling, and disposing of solid wastes (24). For the management of healthcare waste, the Department of Health's (DOH) Revised Health Care Waste Management Manual (2005) consolidates and operationalizes a number of laws, notably the Hospital Licensure Act (RA 4226), the Code of Sanitation of the Philippines (PD 856) and the Toxic Substances, Hazardous, and Nuclear Waste Control Act of 1990 (RA 6969), to govern the management of various types of healthcare wastes–infectious waste, sharps, pharmaceutical wastes, genotoxic wastes, chemical, and radioactive wastes among other typologies (25). During the pandemic, the Philippine government also issued new policies and protocols to guide the management of additional waste generated (6), following guidelines and standards developed by international institutions such as the World Health Organization (WHO).

However, the mere existence of legislation does not necessarily translate to its comprehensiveness and adequacy, nor does it ensure effective enforcement and compliance (26). The rapid development, rollout, adaptation, and implementation of these policies may leave vulnerabilities in the waste management chain and even pose further risks to the public's health, especially in the absence of routine policy review and adaptation (27). Critically, the applicability of pre-existing waste management policies to the COVID-19 pandemic also remains an inquiry yet to be addressed.

There remains to be a dearth in public health literature assessing country-level, waste management policies as applied to the pandemic context. Existing studies have focused thus far on assessing the inventory impacts of medical waste management (28) and municipal solid waste management (29). Domingo and Manejar (30) conducted a recent analysis of regulatory policies on waste management in the Philippines; but only briefly discussed the applicability and effectiveness of these policies to the distinct waste management circumstances brought about by the pandemic.

This study is first of its kind in critically identifying policy gaps and potential implementation challenges for managing healthcare and domestic wastes, within the context of the Philippines' ongoing response to the COVID-19 pandemic. The contextually-relevant results generated by this research, and the criteria-based approach for policy analysis, stands to inform ongoing policy development and adaptation in the country, and importantly, should drive political decision-making and the mobilization of the necessary initiatives and resources for effective policy implementation. This research also provides policy insights and recommendations that may be adopted not only in the local context but prove useful in informing policy development initiatives undertaken by other developing countries faced with similar challenges. Stable legal and institutional bases are critical not only in managing the current waste challenges presented by the pandemic, but also in remaining resilient in the face of future waste crises resulting from public health emergencies (31).

## Methods

We employed a mixed-methods, policy content analysis approach that involved two steps. First, categorical content analysis was done using the Cradle-to-End-of-Life (CTEOL) framework (32) to determine if the included policies for review accounted for all stages of the waste management cycle from production to end disposal, and for three categories of waste (i.e., hazardous healthcare waste, general domestic waste, and household healthcare waste). Step 2 of the content analysis involved utilizing a unified set of criteria adapted from frameworks developed by Rütten et al. (33) and Cheung et al. (34), which evaluated the included policies for sound formulation and potential for effective implementation. Further elaboration of the research methods used can be found in the succeeding subsections.

### Identification of policies

In order to trigger the policy review, a consultative process was first employed to generate a comprehensive and current list of policy documents for inclusion in the study. A database search was coordinated and conducted jointly with internal key informants from the Department of Health (DOH), the Department of Environment and Natural Resources (DENR), and the Department of Interior and Local Government (DILG). Keywords used were: “COVID-19,” “waste,” “waste management,” “healthcare waste,” ”infectious waste” “hazardous waste” “household waste,” “municipal waste,” “solid waste”. We excluded documents that did not contain specific provisions on waste management and those that have already been superseded by Republic Act 9003 or the Ecological Solid Waste Management Act of 2000, and RA 6969 or the Toxic Substances, Hazardous, and Nuclear Waste Control Act of 1990. These core documents provide active policy guidance on the management of municipal waste (including household and community waste) and hazardous waste (such as those generated in healthcare settings), respectively. The policy list generated from the keyword search and screening process was validated and revised with a round of consultations from at least three key informants each from DOH, DENR, and DILG.

The final list included fifty (50) policy documents–both pre-existing and released during the COVID-19 pandemic–that directly influenced the management of COVID-19-related waste generated in the healthcare, household, and community settings. All included policies are national-level issuances that govern all territories in the country, and are thus applicable for implementation from the national, subnational, provincial and local levels.

Using conventions of the Philippine legal system, documents were initially and broadly classified based on level of enforceability (35): [1] laws, [2] implementing policies, and [3] technical guidelines. Laws pertain to the Constitution and legislative statutes such as Republic Acts (Table 1). Implementing policies include executive orders (EO), implementing rules and regulations (IRRs), administrative orders (AO), department orders (DO), and memorandum circulars (MC). These implementing policies, which are founded on already existing laws, are those created to guide programs and administer offices. Technical guidelines include training guides, clinical practice guidelines, operations manuals, and best practice recommendations.

**Table 1:** Classification of policies analyzed in the study.

**Classification**	**Subclassification**	**Number of policies included**
Laws	Republic Acts (RA)	3
Implementing policies	Administrative Orders (AO)	5
	Joint Administrative Orders (JAO)	1
	Department Memorandums (DM)	15
	Memorandum Circulars (MC)	8
	Department Circulars (DC)	5
Technical guidelines	Department Administrative Orders (DAO)-including Implementing Rules and Regulations (IRR) and operational manuals	9
	Resolutions, national plans, other manuals	4
	Total	50

### Policy analysis

A policy analysis team was organized, composed of four Filipino authors, an independent waste management expert, and an independent healthcare waste management expert. Included policies were equitably distributed between the policy analysis team for an initial round of individual content analysis to generate preliminary results. Intercoder reliability for the categorical content analysis (Step 1) was determined at an average of 89% (CI: 84–93) and interrater reliability for the criteria assessment (Step 2) was computed at an average of 84% (CI: 81–88). Inter-rater bias was further minimized by conducting weekly consensus-building meetings, until concurrences in ratings were reached. The findings of the content analyses were then validated through a series of four consultative sessions with relevant policy-making units in the DOH and DENR, and with experts in public health and waste management.

Content analysis of included policies involved two steps. First, the Cradle-to-End-of-Life (CTEOL) Framework by Vozzola (32) was used to assess the applicability of the policies and their underlying provisions to each stage of the waste management life cycle. CTEOL assessments have been extensively used in previous literature to understand the possible environmental impacts of healthcare products and processes during the life of the product (36–39). Applying a lifecycle approach to analysis of waste management policies therefore renders a perspective that considers whether these impacts are accounted for (and regulated) from the time a commodity is produced until it is finally disposed of as waste (32).

**Figure 1:**
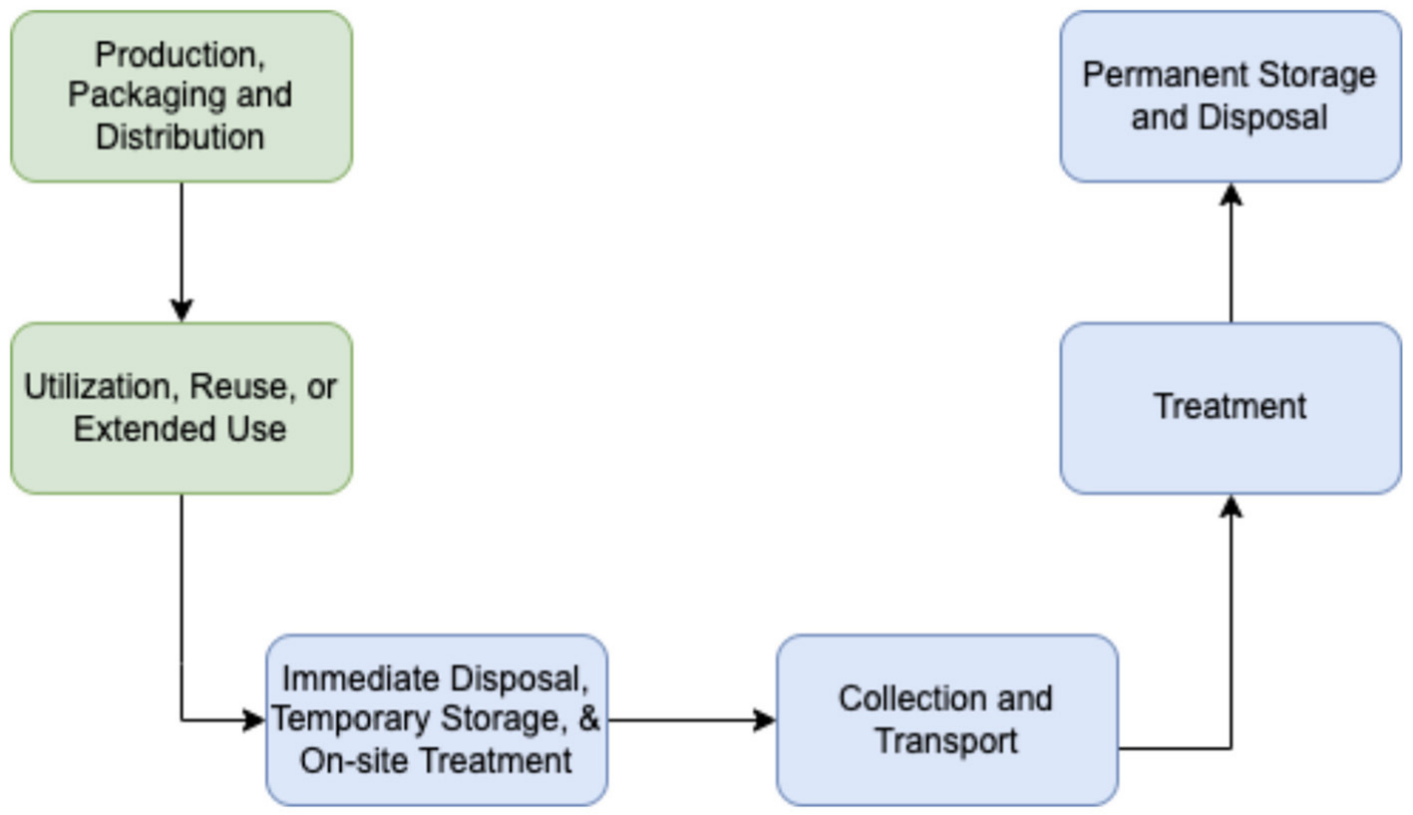
Cradle-to-end-of-life (CTEOL) framework for analyzing COVID-19 waste management policies.

Figure 1 demonstrates the stages in the cycle considered in the CTEOL assessment of COVID-19 waste management policies, reflecting when the product is still a commodity (in green) and when it is considered waste (in blue).

The fifty (50) policies included in the study, including the specific provisions in each policy, were sorted, and tabulated based on the stage(s) within the life cycle covered by the policy.

1. *Production, packaging, and distribution of commodities* covers policy provisions or guidelines that affect production or regulate packaging and distribution (e.g., guidelines on materials used for production, policies on the use of plastic packaging).
2. *Utilization, reuse or extended use* covers policy provisions or guidelines for using, reusing, or extending the utility of the product (e.g., guidelines for rational use and reuse of PPEs).
3. *Immediate disposal, temporary storage and onsite treatment* covers policy provisions or guidelines for waste segregation, storage, and treatment within facilities or settings (e.g., color-coded segregation, designating a specific area for infectious waste).
4. *Collection and transport of waste* covers policy provisions or guidelines for proper handling, management, and regulation of waste during collection and transport from the source to the treatment facility or permanent disposal site (e.g., collection and transport schedule, safety guidelines for waste handlers, separate trucks for infectious waste).
5. *Treatment of waste* covers policy provisions or guidelines for the regulation for treatment and disposal facilities, and proper and appropriate methods of treatment.
6. *Permanent storage and disposal of waste* covers policy provisions or guidelines for the regulation of permanent storage and disposal facilities, and proper and appropriate methods of disposal (e.g., incineration, landfill).

Secondly, the internal validity of waste management policies in terms of sound formulation and potential for effective implementation were then evaluated against a set of criteria adapted from the policy analysis frameworks developed by Rütten et al. (33) and Cheung et al. (34). Compared to other policy analysis frameworks, the criteria used by Ret al. and Cheung et al. focus on the goals, resources, obligations and opportunities that form the determinants of effective policy formulation and implementation, and have thus far demonstrated utility in other literature, especially for the assessment of health promotion policies (39, 40).

Building on these criteria and supported by face validation with experts and policy makers (acknowledged at the end of this paper), and further literature review (40–42), the policy analysis team utilized 12 policy analysis criteria as follows: 1) Policy Accessibility, 2) Strength of Policy Background, 3) Clarity of Goals, 4) Provision of Financial Resources, 5) Provision of Human Resources, 6) Organizational Capacity-building, 8) Contingency and Sustainability, 9) Monitoring and Evaluation, 10) Public Opportunities, 11) Equity, and 12) Obligations. The underlying rubrics for each criteria are presented in Table 2.

**Table 2:** Modified criteria for analyzing COVID-19 waste management policies.

**1. Accessibility**
1. A soft copy of the policy document is readily available and easily accessible online 2. A hard copy of the policy document is readily available and easily accessible, or at the very least its physical location is made known to the public
**2. Policy background**
1. The scientific grounds of the policy are established 2. The legal grounds, i.e., Republic Acts and/or Executive Orders, are established 3. The policy and its goals are drawn from a rigorous and conclusive review of literature, such as international best practices and relevant local statistics 4. The source of the policy is explicit
a. Authority (experts and/or primary literature such as books and journals) b. Qualitative or quantitative analysis c. Deduction, where premises that have been established from authority, observation, experience, or all three)
**3. Goals**
1. The goals are explicitly stated 2. The goals are concrete enough to be evaluated (quantitatively, qualitatively, or both) 3. The goals are clear their intent and mechanisms 4. The actions center on improving the health of the population 5. The goals and outcomes contribute to the intended health outcomes
a.There is external evidence for logically drawing health outcomes b.There is internal validity for logically drawing health outcomes
**4. Financial resources**
1. Costs from start to end are explicitly mentioned and accounted for. 2. Means and mechanisms to pay for resources, goals, actions, and outcomes are stated 3. Financial resources are listed and their allocations are clearly stated
**5. Human resources**
1. The policy accounts for and assigns point persons for each of its activities
a.There is enough personnel to carry out the policy b.The policy specifies the roles and obligations of each personnel/implementer for each activity
2. The action is part of policy implementer's existing duties
**6. Organizational capacity**
1. The organization and its partners have necessary and sufficient resources and capabilities to carry out the policy from start to end 2. There is capacity building through adequate training, supervision, and technical assistance in order to carry out activities of the policy
**7. Contingency and sustainability**
1. The policy takes into consideration contingencies by having measures and mechanisms to deal with disasters, pandemics, and other emergencies, and their aftermath 2. The policy is sustainable and is feasible and applicable to different contexts during the short-term and long-term recovery period after contingencies occur
**7. Contingency and sustainability**
3. The policy renders itself sustainable enough to mitigate and prevent future environmental health and public health risks
**8. Monitoring and evaluation**
1. The policy indicates clear, sufficient, and specific criteria and mechanisms for monitoring and evaluation 2. The policy nominates an independent body to perform the evaluation 3. The policy identifies outcome measures for each objective 4. The data for evaluation is collected before, during, and after the policy is introduced or implemented 5. Follow ups take place after a sufficient period of time 6. Factors other than the policy that could have produced changes are identified
**9. Public opportunities**
1. The policy takes into consideration the public's current level of awareness on the policy itself, its context, and issues surrounding it 2. The policy has mechanisms to build the awareness of the public, the stakeholders involved, and the sectors affected by the policy 3. The general public and its various sectors support the action and provide long-term support 4. Multiple stakeholders are involved 5. Primary concerns of affected sectors/individuals are taken into consideration
**10. Equity**
1. 1. The policy is feasible, applicable to the contexts of marginalized sectors, and its mechanisms are accessible to said sectors.
a. Low-income classes and those unemployed b. Women and the LGBT+ Community c. Indigenous Peoples (IPs), Indigenous Cultural Communities (ICCs), and ethnolinguistic groups, Internally Displaced Persons (IDPs) and refugees, and other geographically isolated persons d. Persons with Disabilities (PWDs) e. Prisoners f. Persons in conflict areas g. Children
2. The policy takes into consideration differences in impacts on different sectors a. The policy does NOT pose disproportionate risks to certain marginalized populations b. The policy does NOT place disproportionate or unequal benefits/access toward certain groups over others
3.The policy is grounded on the reality that certain populations are currently suffering under a greater deal of difficulties compared to others, therefore the policy has mechanisms to address the unequal impacts its implementation will bring
**11. Obligations**
1.The policy is compelling enough to ensure compliance of the implementers, stakeholders, and affected populations a. Scientific results (quantitative, qualitative, or both) are compelling for action b. Legal bases are compelling for action
**11. Obligations**
2. The policy lists rewards/sanctions if activities are not conducted/implemented 3. The policy lists rewards/sanctions for spending allocated resources on activities unrelated to the policy

Tabulations were made based on the extent to which the policy fulfills each criteria. Criteria were considered “Fulfilled/Strong” if all the mentioned criteria were addressed, “Room for improvement” if some of the needed aspects were unaddressed and “Not fulfilled/Weak” if none or nearly none of the criteria were addressed (35).

## Results and discussion

### Cradle-to-end-of-life analysis

Content analysis using the Cradle-to-End-of-Life (CTEOL) framework demonstrated that every stage of the waste management life cycle is already covered by existing laws, government policies, and guidelines, as shown in Table 3.

**Table 3:** Applicability of policies and provisions at each stage of waste management life cycle.

**Modified CTEOL stages**	**Applicable policies per stage**	**Applicability of policies per type of waste**			**Applicable provisions per stage**	**Applicability of provisions per type of waste**		
		**To hazardous healthcare waste**	**To household healthcare waste**	**To general solid waste**		**To hazardous** **Healthcare waste**	**To household healthcare waste**	**To general solid waste**
Production, packaging, and distribution	25	23 (92%)	4 (16.00%)	11 (44.00%)	135	106 (78.52%)	7 (5.19%)	22 (16.30%)
Utilization, reuse, and extended use	30	29 (96.67%)	11 (36.67%)	13 (43.33%)	250	135 (54.00%)	46 (18.40%)	69 (27.60%)
Immediate disposal, temporary storage, and on-site treatment	38	33 (86.84%)	7 (18.42%)	12 (31.58%)	224	148 (66.07%)	20 (8.93%)	56 (25.00%)
Collection and transport	30	26 (86.67%)	2 (6.67%)	7 (23.33%)	101	77 (76.24%)	2 (1.98%)	22 (78.00%)
Treatment	19	17 (89.47%)	1 (5.26%)	5 (26.32%)	46	35 (76.09%)	1 (2.17%)	10 (21.74%)
Permanent storage and disposal	29	23 (79.31%)	5 (17.24%)	7 (24.14%)	110	58 (52.73%)	12 (10.91%)	40 (36.36%)

Further content analysis of the fifty (50) policies revealed comprehensive coverage on the management of hazardous healthcare waste and general solid waste across life cycle stages. However, there remains a dearth in laws regarding the management of household healthcare waste, a new and unique waste category that emerged during the pandemic. Broadly defined, household healthcare waste pertains to waste generated by individuals suspected or confirmed with COVID-19, who are currently isolating or quarantined at home, and may include “contaminated and non-contaminated personal protective equipment, expired and discarded medicines, injection needles and other sharps, and self-administered testing kits among others” (43). Given the novelty of this waste category, the included policies in this review have not yet provided any official policy definition or criteria for what constitutes “household healthcare waste” in the Philippine context. Though existing policies may seem to apply to this new category, they are prone to be subjected to different interpretations if left without specific definition or policy guidance.

The policy analysis and gaps identified across each lifecycle stage are discussed further in the succeeding subsections.

#### On production, packaging, and distribution

Policy provisions and guidelines included in this stage are those that affect production or regulate packaging and distribution (e.g., guidelines on materials used for production, regulations for distribution, etc.) For healthcare products that will eventually be converted to hazardous healthcare waste, twenty-three (23) policies (46%) gave emphasis to increasing production and procurement, especially for single-use commodities (including PPEs) and for dedicated transport vehicles required for managing infectious patients and healthcare waste. A circular from the Food and Drugs Administration (FDA Circular 2020-014) also provided manufacturing guidelines for local PPE production to ensure safety and quality, but provided no commentary on the deleterious impacts of utilizing single-use plastics (SUP) for commodity production and packaging. At the same time, it also did not provide any recommendation on the use of environmentally acceptable materials for production.

Four (4) policies (8%) were found applicable to the production and procurement of healthcare products used in household settings, instructing the acquisition of more PPEs and disinfectant supplies for proper handling of hazardous waste from the Department of Health and Department of Interior and Local Government. DOH DM 2020-0270-A and DOH-DILG Joint AO 2020-0001 ensure that waste facility personnel must be provided with appropriate PPE. DILG MC 2020-147 and DOH AO No. 2020-0015 mandated the availability of disinfectants and hand sanitizers in public establishments and all waste transport vehicles. However, the lack of policy guidance on a needs-based mechanism for equitable production and distribution of these commodities may lead to overproduction and over procurement. Ordinary citizens are most susceptible to indiscriminate use of PPEs, resulting not only in incorrect handling and disposal, but also supply shortages in healthcare facilities where these are most needed (44, 45).

For general products that will eventually be converted to general solid waste, eleven (11) policies (22%) recommended increased production for necessary general supplies such as linens, bleach, towels, and raw materials, but there were no policies that covered or regulated the production and packaging of SUPs. The Ecological Solid Waste Management Act of 2000 (ESWMA) or RA 9003 stipulated that the government should promote recyclable products and discourage products that use non-environmentally acceptable products and packaging (NEAPP). Though passed into law over 20 years ago, progress on the NEAPP list remains to be slow and generally unenforced (46). Not a single product was listed until 2021, when plastic soft drink straws and coffee stirrers were recently added (47). Local civil society groups and non-government organizations (NGOs) have since advocated for the list's urgent expansion to include other SUPs that have been found to significantly contribute to the mounting plastic problem during the pandemic, including plastic bottles, cups, takeout containers, styrofoam food containers, sachet and other multi-layered plastic packaging (48).

Without policy regulations to promote the circular economy for SUPs, the drastic increase in its production and demand would inevitably lead to tremendous plastic pollution, a significant portion of which is flushed down into water ecosystems (49). Plastic waste and its degradation products (e.g., microplastics) are easily ingested by fish and other bio-marine organisms, which in turn re-enter the human food chain and cause chronic human health problems (49, 50). The global, amplified concern for plastic pollution secondary to the use of PPEs, especially single-use face masks, has stimulated research for sustainable materials for production such as bio-based plastics (49, 51), but has yet to be mainstreamed in resource-limited settings such as the Philippines.

#### On utilization, reuse, and extended use

Policy provisions or guidelines included in this stage pertain to those that govern the use, reuse, or extension of the utility of products. For products used in healthcare settings, twenty-nine (29) policies (60%) had provisions and guidelines on the rational use of PPEs (single-use PPEs, masks, and face shields) and home testing kits (including rapid antigen and antibody tests). Eleven (11) of these policies (22%) were found applicable to household settings, with three (3) of these specifically applied to persons isolating at home or placed on preventive quarantine. DOH Department Memorandum No. 2020-0105 instructs isolating or quarantined patients on proper mask usage and its discard after one-time use. DM 2020-0090 described how utensils and dishes must be thoroughly washed after use and may be reused thereafter. DOH-DILG Joint AO 2020-0001 recommended disposable paper towels to dry hands, use 60% alcohol-based sanitizers when soap and water are not available, and to clean frequently touched surfaces with bleach or detergent.

Unfortunately, there was a notable lack of unified guidelines on the rational use of home-testing kits and for the safe reuse of PPEs at the household level. The absence of such policy guidance may result in hoarding, unregulated use, and improper recycling of single use PPEs, eventually leading to excessive waste generation and improper waste management. This has been documented in the Philippines especially during COVID-19 surges, when there has been observed panicked marketplace behavior (52). Mandatory but unguided use of disposable facemasks for all people in public spaces was estimated to generate as much as 41 million pieces of masks (150,000 tons of plastic waste) in the Philippines daily (5, 6, 39).

For products that will eventually be converted to general solid waste, thirteen (13) policies (26%) provided guidelines for the use of disinfectants and other general supplies. DOH DM 2020-0157 mandated the timely disinfection of all public places, including public establishments, roads, and pavements, but lacks further guidance on the safe and proper application of such chemicals. Oxidative chemicals used in disinfection have their own environmental and public health ramifications. Chlorine (NaClO), the cheapest and most commonly used disinfectant, generates toxic by-products that are harmful to marine ecosystems and can persist in environments longer than chlorine (53). Regular use of other common disinfectants like ammonium and bleach are also known to have negative health impacts (54), with links to chronic pulmonary disease among healthcare workers (54) and asthma in young children with early exposure at home (55).

#### Immediate disposal, temporary storage, and on-site treatment

Policy provisions and guidelines included in this stage include those affecting disposal, waste segregation, storage, and treatment within facilities or settings. For hazardous healthcare waste, thirty-three (33) policies (66%) emphasized the segregation of waste, decontamination, disinfection, sterilization, and flow of patients in hospitals, vaccination centers, and quarantine centers. Four (4) policies specifically mentioned the use of on-site waste storage through concrete vaults or septic tanks (DOH DC 2020-0219, DC 2020-0191, DILG MC 2020-052, and the National Vaccination Plan). DC 2020-0191 and DOH-DILG Joint AO 2020-0001 encouraged on-site disinfection to allow healthcare facilities to have more control over their waste disposal processes and costs.

DOH DC 2020-0049 laid down the proper decontamination, disinfection, and sterilization practices for various types of healthcare items. It also listed the different methods of on-site treatment available for different levels of disinfection and the corresponding processes to be followed. During the pandemic, healthcare facilities were encouraged to use autoclaves and other technologies for large volume final disposal arrangements and environmental control (New Normal in All Health Facilities Policy Policies, DOH DM 2020-0208, National Vaccination Plan). Autoclaving remains a popular method for disposal in low-income countries due to scalability, applicability to up to ninety percent (90%) of hospital waste, and comparatively low capital costs (56–59).

Wastewater management was given further legislative guidance with detailed guidelines mandated by DC 2020-0191. Uncoupling hospital wastewater management from municipal wastewater is important in preventing the spread of antimicrobial-resistant bacteria, which can spread antibiotic-resistant disease and affect aquatic ecosystems when ejected into wastewater systems (60, 61).

For general solid waste, twelve (12) policies (24%) guided the immediate disposal and segregation of domestic waste, recommending the use of color-coded bins, practice segregation at source, proper labeling, and safe handling of waste. The ESWMA or RA 9003 and technical guidelines from both DOH and DILG gave comprehensive guidelines on receiving and sorting waste for recyclable resource recovery to ensure it is in the most efficient, environmentally sound manner.

For household healthcare waste, seven (7) policies (14%) were found applicable to its immediate disposal and segregation but were non-specific, referring to all hazardous waste regardless of the setting where it is generated. Non-specific policy guidance and provisional support dedicated to the segregation of household healthcare waste at the source may lead to improper mixing with general solid waste, and public health risks from this category may inadvertently cascade down the succeeding waste life cycle stages, such as collection, transport, and permanent disposal (62).

#### Collection and transport

Policy provisions and guidelines analyzed in this stage pertain to the proper handling, management, and regulation of waste during collection and transport from the source to the treatment facility or permanent disposal site. When handling healthcare waste for collection and transport, twenty-six (26) policies (0.52%) instructed that great care must be given to prevent mixing of waste and exposure of staff during transport. Waste containers must be color coded and tightly sealed after collection. Waste receptacles should be emptied and collected at regular time intervals. Transport should also be done during off-hours and in the safest, most efficient way, ensuring the welfare of the healthcare workers, patients, and formal waste service providers.

For the collection and transport of household healthcare waste, two (2) policies (4%) were applicable but nonspecific to this waste category (DOH DM 2020-0270-A, DILG MC 2020-147). Both issuances indicated that hazardous waste must be kept in a separate container during collection and transport. However, the lack of specific implementing mechanisms for collecting and transporting household healthcare waste during the COVID-19 pandemic poses a problem of improper mixing with general domestic waste, which may result in further spread of disease amongst waste workers and collectors (45, 59).

Notably, informal waste pickers, who are vital components of waste collection and resource recovery from household settings in the Philippines, were not mentioned in any of the policies reviewed. Exclusion of this sector from policies is a public health issue and without regulatory safeguards from occupational hazards, this sector is left highly vulnerable (63–65). Lack of legislative support for informal waste pickers is also a missed opportunity to not only protect the environment, but to also help reduce poverty in this sector (65). Payatas, Quezon City, the biggest landfill scavenging site in the Philippines, provides sustainable livelihoods to three thousand (3,000) informal waste pickers alone (64).

For general solid waste collection, seven (7) policies (14%) gave specific guidelines for vehicle permit requirements and collection materials (bags, carts, ramps) needed for easy collection and transport of domestic waste. The ESWM Act of 2000 was very detailed in its provisions for the collection and transport of domestic waste for optimal resource recovery. However, no further policy guidance has been executed for increasing frequency and capacity for waste collection, transport, and materials recovery to account for the substantial rise in general solid waste generation during the pandemic.

#### Treatment

Policy provisions and guidelines in this stage regulate treatment and disposal facilities, and proper and appropriate methods of treatment. Seventeen (17) policies (34%) guided the treatment of hazardous healthcare waste in disposal facilities. In the Procedural Manual Title III of DAO 92-29 “Hazardous Waste Management” DENR AO 36 Series of 2004, treatments recognized by policy for this waste category include physicochemical transformation treatments (i.e., neutralization, oxidation, reduction of waste acid, waste alkali, or waste solution), thermal treatments (i.e. autoclave, microwave, and sterilization), decomposition, immobilization, encapsulation, polymerization, solidification, melting, thermal decomposition, rinsing, and reprocessing. A list of waste materials unacceptable for co-processing were also defined (Amendment on Some Provisions of DAO 2010-06). Additionally, a manifest system was created to ensure that the collected infectious wastes are properly recorded, treated, and disposed, as written in the Guidelines on the Management of COVID-19 Related Health Care Wastes (DILG MC 2020-147).

Interestingly, a policy contradiction was identified with a recent issuance (DILG MC 2020-147) allowing incineration of healthcare wastes as alternative modes of treatment and disposal, stating that COVID-19 related wastes must be “properly treated with available technologies (i.e., sterilization, thermal processing like pyrolysis and gasification, incineration, etc.).” This provision bypasses two landmark policies (Clean Air Act and Ecological Solid Waste Management Act) which disallow all forms of incineration even under emergency situations such as the COVID-19 pandemic.

For household healthcare waste, only DILG MC 2020-147 was found applicable but still non-specific to this category, mandating the treatment of all COVID-19 related waste using appropriate, available technology. However, without enforced guidelines for the segregation of household healthcare waste at source, this waste category would bypass appropriate waste treatment, be immediately sent to landfills and cause mixed contamination with general domestic waste, minimizing opportunities for resource recovery and increasing risks for disease transmission down the waste management chain (66).

For general solid waste, five (5) policies (10%) had provisions for the processing and treatment of domestic waste, but no specific technologies were indicated. New technologies for treatment were also neither explored nor incentivized, particularly in the interest of exploring best available technologies (BAT) to unburden sanitary landfills in the country which are currently operating beyond absorptive capacity.

#### Permanent storage and disposal

Policy provisions and guidelines in this stage pertain to the regulation of facilities designated for permanent storage and disposal, as well as the proper and appropriate methods of disposal. For hazardous healthcare waste, twenty-three (23) policies (46%) provided guidelines and proposed technologies for their separate end storage and disposal. Infectious waste was mandated to be sent to landfills with dedicated disposal spaces (DENR AO 1998-50, DOH DC 2020-0049, DOH DM 2021-0031, DM 2020-0170, and DC 2020-0191). Interestingly, a DENR report showed that only 29% of healthcare waste from April to July 2020 was properly treated and disposed of in landfills due to the lack of capacity to accommodate the sudden surge of healthcare waste (67). This may pose a significant public health risk as healthcare waste can be a major source of chemical pollution and cause illnesses such as liver diseases, cancer, and the further spread of COVID-19 especially among communities and livelihoods adjacent to disposal sites (68).

For household healthcare waste, five (5) policies (10%) were found applicable to this category, but still non-specific. This gap in policy poses a problem as unsegregated household waste cannot be co-processed and would be permanently disposed of in landfills, which cannot be open dumps. Unsanitary dumping of hazardous, untreated waste in landfills can cause possible viral transmission amongst informal waste pickers and the leaching of harmful chemicals into the environment (67). Space and logistic constraints from overcrowded landfills may also eventually lead to open dumps, threatening nearby aquatic and terrestrial biota (66, 67).

For general solid waste, seven (7) policies (14%) gave provisional support for sanitary landfill sites and requirements. These policies include geographical and environmental considerations in choosing a site and building facilities. However, there is a scarcity of new landfill sites and limited logistics to accommodate increasing amounts of waste, especially in provincial areas (67, 69). This has historically led to open dumping and incineration of as much as sixty percent (60%) of waste nationwide (67, 69).

### Criteria assessment for analyzing public health policy documents

In order to assess internal validity and potential effectiveness during policy implementation, the fifty (50) policies were also evaluated against a set of 11 criteria adapted from the policy analysis frameworks of Rütten et al. and Cheung et al. (34, 35) and reflecting the Philippines' policy implementation context. Criteria were considered “Fulfilled/Strong” if all the mentioned criteria were addressed, “Room for improvement” if some of the needed aspects were unaddressed and “Not fulfilled/Weak” if none or nearly none of the criteria were addressed.

Table 4 summarizes the result of the criteria analysis.

**Table 4:** Criteria assessment on the internal validity of waste management policies.

**Criteria**	**Fulfilled or strong**	**Room for improvement**	**Not fulfilled or weak**
*N* = 50 policies			
Accessibility	50 (100%)	0 (0%)	0 (0%)
Policy background	27 (54%)	20 (40%)	3 (6%)
Goals	34 (68%)	11 (22%)	5 (10%)
Financial resources	6 (12%)	11 (22%)	33 (66%)
Human resources	28 (56%)	13 (26%)	9 (18%)
Organizational capacity	13 (26%)	24 (48%)	13 (26%)
Contingency and sustainability	11 (22%)	13 (26%)	26 (52%)
Monitoring and evaluation	17 (34%)	9 (18%)	24 (48%)
Public opportunities	14 (28%)	23 (46%)	13 (26%)
Equity	4 (8%)	19 (38%)	27 (54%)
Obligations	21 (42%)	18 (36%)	11 (22%)

The criteria assessment demonstrated that majority of the policies required further improvement across all criteria except for “Accessibility.” It is also noteworthy that a significant number of policies weakly fulfilled the criteria on provision of adequate financing, ensuring organizational capacity building, providing for contingencies and sustainability, conduct of monitoring and evaluation, encouraging opportunities for public participation, and promoting equity.

Relevant findings on the fulfillment of policies for each criterion are discussed in the succeeding subsections.

#### Accessibility

In this study, accessibility was assessed on the availability of the policy, either as an online or physical document in a platform that is accessible to policy implementers, end users and the public. All of the policies included in the study were found in the official databases of DOH and DENR and are publicly accessible online in official government websites. However, it also stands that online policy databases in government websites are not frequently accessed due to ineffective policy marketing and dissemination mechanisms (70), compounded by unreliable internet connectivity in many parts of the country (71, 72). As such, the extent of actual accessibility, specifically to implementers and to the public in general, and particularly during the pandemic, remains unknown.

Moreover, a review of the posting of the policies revealed that the release of new or amended policies during the pandemic was staggered and fragmented, i.e., they were usually released reactively at different periods over the pandemic, and separately by different policy authorities. The absence of a single platform where policy updates may be accessed stands as a barrier to ensuring common policy understanding and harmonized implementation.

#### Policy background

A document that has a strong policy background is one where scientific, legal and authoritative grounds are clearly established. Sources must be explicitly cited and where deductions are made, premises must be founded on authority, technical expertise, or direct observation (34). Among the fifty (50) policies, twenty-three (23) of them (46%) were rated as either needing improvement or weakly fulfilled. While all of the policies provided supporting legal literature as foundation, very few of the policies made purposeful use of supporting statistics, findings from peer-reviewed scientific literature, or recommendations from expert consultations to form a “sound” basis for establishing the policy background. Among the policies found to have good policy background, statistics and sources were referenced from the World Health Organization, the US Center for Disease Control and Prevention, and from presentations of monitoring data provided by national government authorities.

#### Clarity of goals

For a policy to be properly implemented, its goals and objectives must be explicitly identified and mapped out (34). A majority of the policies (68%) evaluated had clearly stated and explicit goals, which enables such policies to have a precise direction. The goals presented were concrete enough to be evaluated objectively and were definite with their mechanisms and intended outcomes. Of the remaining 32% of policies needing further improvement on this criteria, the goals were found to have limited internal and external consistency in guaranteeing that larger health and environmental outcomes may be derived from policy goals and outcomes. For instance, policies proposing certain waste handling and storage methods failed to provide further evidence on the relative effectiveness of such options in reducing fomite transmission. Policies that laid out certain waste treatment methods also did not disclose the potential environmental impacts of these methods (e.g., incineration, chemical decontamination).

#### Financial resources

A total of 44 policies (88%) neglected to provide a breakdown of financial resources, or only did so in a vague, passing manner. For example, DILG MC 2020-147 mandated that at the municipality level, “as much as practicable,” households must be provided with temporary storage bags and bins. It also stated that all local units must use available technologies for monitoring and treating waste. On the other hand, DOH AO No. 2020-0015 mandated the “Provision of support for essential workforce (ex: financial, lodging, shuttle, food, etc.)” without much elucidation or obligation of financing sources and agents.

Furthermore, instead of recommending an increase in budget allocation for waste management, policies would call for the provision of financial resources only in a generic way. The National Vaccination Plan simply stated that authorities should “Facilitate the budget for the campaign's operations” and “Develop a budgeted cold and logistics plan.” AO2021-0005 also only mentioned that there is a need “To develop a cold chain and logistics plan and provide a budgetary plan to the COVID-19 vaccine clusters for cold chain and logistics management.”

These are illustrations of how policies merely state materials and processes needed without listing the budgetary and investment requirements needed to achieve them. In order for a policy to be truly effective, all costs and their allocations from start to end must be explicitly accounted for. This includes all means and resources to pay for each of the goals, actions, and outcomes (34).

#### Human resources

A policy must also account for and assign dedicated personnel for implementing and monitoring its proposed activities and mechanisms. This is to ensure that there is enough personnel and absorptive capacity to carry out the policy, and that specific roles and obligations are delineated (34). Twenty-eight (28) of the fifty (50) policies (which accounts for 56%) accounted for personnel needed and outlined specific roles these personnel are accountable for. The ESWM Act also delineated roles at the different levels from the national, regional, provincial, city/municipality, and individual levels. The city/municipal level is responsible for the collection and transport of wastes. The municipality level is responsible for recovery, recycling, and reuse of wastes. Cities and municipalities may form partnerships and arrange contracts with the private sector for supplementing these roles. The individual or the source is responsible for sorting and segregating wastes. Interestingly, none of the policies recommended the provision of additional human resources for waste management despite increasing and shifting workloads ushered in by the COVID-19 pandemic. However, one policy ensured that health facilities without service providers, particularly for waste transport or disposal, would be supported by relevant government agencies, but without specifying distinct roles and obligations.

#### Organizational capacity-building

Organizational capacity-building includes policy provisions for policy cascading, training and technical capacity-building, and provision of logistical and technical support for the ground implementation of waste management efforts. This criterion ensures that the implementing agencies and bodies have the awareness, readiness, absorptive capacity, and sufficient resources to bring the policy into fruition (34).

Thirteen policies (26%) strongly fulfilled this criterion while another 13 (26%) were unable to do so. A common theme across the policies was the provision of protective gear such as masks, gloves, face shields, and cleaning and disinfection for workers. NSWMC Resolution 1364-2020 also highlighted the need for waste disposal personnel to be informed about the waste they are handling and to be protected through precautionary measures such as wearing of PPEs and maintaining proper distance from the waste. Related guidelines were also found available for handling toxic chemicals and spill control. Many of the policies, while geared toward capacity-building, had nonspecific provisions on the implementation resources and technical capability building needed to ensure full absorptive capacity of waste management processes. As an example, none of the policies covered providing technical and logistical support for on-site storage and treatment of healthcare waste in lower level facilities who may not have existing facilities and capacities for such, especially those in the public sector.

Even more fundamental, none of the policies lacked guidelines as to how provisions, mandates, and responsibilities are to be cascaded to local implementers, especially in the context of the Philippines' devolved governance setup where the local governments have the mandate to implement waste management policies within their jurisdiction.

#### Contingency and sustainability

The Philippines experience made it apparent how public health emergencies such as extreme surges in the number of COVID-19 cases may coincide with other disease outbreaks (e.g., dengue and leptospirosis) and the occurrence of climate-related disasters (e.g., typhoons, earthquakes), which taken altogether, may synergistically overwhelm public health and waste management systems and easily derail the implementation of set mechanisms established by policies and guidelines. A policy that takes into consideration contingencies lists measures and mechanisms to deal with and adapt to foreseen and unforeseen circumstances with their aftermath. This includes disasters, co-existing disease outbreaks, and other emergencies (34). The policy must also be sustainable and feasible during short-term and long-term recovery periods, and render itself viable to mitigate and prevent future environmental and public health risks under different contexts (34). Even the WHO, in their Guidance for Contingency Planning (73), underscores the need to develop mechanisms for conceivable threats. This is to minimize potential risks and public health consequences, to prepare plans of action and to ensure provision of adequate resources accounting for these risks. WHO further states that “All plans must be regularly updated based on the evolving risks and environment.”

Only eleven (11) of the fifty (50) policies (22%) satisfied this rubric. The rest of the policies provided no plans on how provisions and implementing mechanisms may be applied or adapted during emergencies, or for situations out of the ordinary. Older waste management policies do consider factors such as managing wastes during rain and leakages, but there was little attention toward the possibility of major calamities and flooding disasters–not uncommon in the Philippines–that may completely derail established mechanisms for waste segregation, collection, storage, and disposal. The idea of contingency plans was mentioned briefly in new policies released during the pandemic, but provided very little detail on the implementing mechanisms of these contingency plans. Protocols for managing malfunctioning equipment, accidents and emergencies were often mentioned as a requirement, but no specific instructions or guidelines were provided in doing so.

There was also little to no mention of mechanisms for the safe continuation of recycling efforts during the COVID-19 pandemic. This would stand to delimit efforts at preventing end disposal facilities from being overwhelmed due to the additional amount of wastes generated during the pandemic. In other countries, recycling network models using a reverse logistics design have since been developed and proposed based on case studies in China (74, 75) and Iran (76, 77), but have yet to be seen in effective practice.

#### Monitoring and evaluation

A policy implementation review is useful to ascertain the effectiveness of policies in terms of its implementation. The policy itself must provide clear, comprehensive, specific, and understandable criteria for its own monitoring and evaluation. Specifically, the following must be stated in the policy: outcome measures per objective, the independent body that will perform the evaluation, and the timeline of evaluation data collection (34). The timeline, which includes follow-ups, must include factors outside of the policy that could have produced changes in the implementation. It is even recommended that waste management plans are analyzed per region, province, city, or municipality, given the devolved nature of the Philippines' public health and waste management systems.

Nearly half or 24 of the policies (48%) analyzed had absent provisions for policy monitoring and evaluation. Only a few policies would designate persons to conduct checks and balances or identify a system of penalties to be imposed. However, the specific indicators, monitoring mechanisms and activities for follow-ups were not indicated.

#### Public opportunities

A policy that is strong in its public opportunities is one that strengthens the public's level of awareness of the policy as well as their participation and engagement in the policy development process (34). Only 14 policies (28%) were able to strongly satisfy this criterion. Most of the policies acknowledge multiple stakeholders to the policy, yet do not make mention of mechanisms to build their awareness nor to consult their perspectives during policy formulation, implementation, and evaluation, signifying a top-down approach. A few policies take into consideration the signs of times surrounding the policy (i.e., needs arising from the pandemic), but fail to account for the context of the stakeholders and affected sectors themselves. Effective policy implementation and assurance of policy compliance is challenging without inclusive public engagement.

#### Equity

Majority of the policies analyzed failed to acknowledge how provisions may differentially affect various sectors. An effective public health policy is grounded on the reality that certain populations may be more impacted–or at least affected differently–by policies than others. They fall under different contexts and may thus have different forms of adapting or ways of applying policies. Therefore, the policy must have mechanisms to address the unequal drivers and impacts that its implementation will bring. It must be feasible and applicable to the contexts of marginalized sectors, and its mechanisms must also be accessible to them. Policies should not pose disproportionate risks to certain sectors while affording disproportionate benefits to others. Key populations identified were (1) low-income classes and those unemployed, (2) women and the LGBT+, (3) indigenous peoples, internally displaced persons, and other geographically isolated persons, (4) persons with disabilities, (5) prisoners, (6) persons in areas of conflict, and (7) children.

With only 8% or four (4) out of fifty (50) policies strongly fulfilling this criterion, equity is the criterion that had the lowest number of policies ranked as “Strongly Fulfilled.” Most of the policies reviewed had no provisions that specifically considered the social vulnerabilities faced by Filipinos today and their lack of alternatives, especially in low-income communities and those in remote, rural areas.

#### Obligations

The final criterion for analyzing public health policy documents is its ability to become obligatory. The policy must be compelling enough to ensure compliance of the implementers, stakeholders, and target populations. This may be done through the provision of rewards and imposition of penalties and sanctions. Twenty-one (21) of the fifty (50) policies (42%) stated penalties for improper or inadequate implementation, and also provided guidelines for their imposition. The policy documents analyzed also indicated that performance metrics were to be reported by groups responsible for monitoring and evaluation, to be later on used as basis for the provision of incentives and/or disincentives. Without such monitoring systems in place, penalties and sanctions are likely to be disregarded and mandates and responsibilities may be foregone.

## Actionable recommendations

### On effective and dedicated management of “household healthcare waste”

The COVID-19 pandemic ushered in a new category of waste: household healthcare wastes. As there was no official definition or criteria for what constitutes “household healthcare waste,” this category was vaguely and nonspecifically accounted for in the policies analyzed. With neither an explicit definition nor policy guidance, managing this new category of waste may be subject to different interpretations. Thus, we recommend that this category be officially recognized during policy formulation to avoid mishandling of hazardous waste from domestic and other non-healthcare settings.

As a next step, policy-backed strategies can be developed to ensure capacity-building programs and adequate public opportunities for individuals, households, and waste handlers dealing with household healthcare waste, so as to minimize occupational health risks and environmental impacts. To ensure enforceability in the context of the Philippines devolved governance system, local government should be mandated by national policy to develop local policies that enable and incentivize household-level segregation and disinfection. These may include local guidelines on specific schedules for collection of household healthcare wastes, and provision of dedicated, color-coded waste receptacles for households with quarantined or isolated individuals as well as dedicated bins for mask waste disposal in public areas. A similar policy on the management of household healthcare wastes has since been executed in Indonesia in 2020, through its Circular Letter on Infectious Waste and Household Waste Management during the COVID-19 Pandemic, however, policy enforcement and compliance remains a challenge (78). To this end, policies that support and finance information campaigns on proper handling of household healthcare waste will not only ensure policy compliance, but also mitigate misinformation and build solidarity.

### On intervening early in the waste life cycle

Strong mandates within pre-existing laws need to be strengthened or adjusted to ensure that waste management strategies during the COVID-19 pandemic do not further overwhelm the waste management chain. One such law is the Ecological Solid Waste Management Act, which already recognizes the relative advantage of strengthening waste reduction and recycling efforts over treatment and disposal in the waste management hierarchy. The observed rise in the production, utilization, and disposal of single-use plastics during the COVID-19 pandemic, in the absence of a suitable policy framework that supports early interventions in the waste management chain, challenges this directive.

To this end, the development and mainstreaming of policies and guidelines for the production and safe utilization of reusable PPEs stands to mitigate further generation of preventable plastic waste, both in healthcare and household settings. However, such issuances must balance cost-effectiveness with potential environmental impacts, since reusable PPEs and other commodities are not without environmental footprint. This may be further supported by guidelines on proper disinfection and extended use of these commodities. The same strategy may be applied for general household products that can be safely reused with proper disinfection. Critically, the list of single-use plastic products included in the Non-environmentally Acceptable Products and Packaging (NEAPP) must be expanded and updated to discourage the use of SUPs for production, packaging and use in both healthcare and household settings.

### On ensuring continuity and adaptability of the waste management chain

The COVID-19 pandemic continues to present complex, dynamic, and evolving challenges to waste management systems and facilities in the Philippines, and as such, policies and guidelines are expected to rapidly and responsively adapt to these evolving challenges over time, if only to maintain service continuity. While the policy analysis identified policy adaptations made at certain stages of the waste management chain, the observed lack of adaptation in guidelines concerning recycling and waste collection presents opportunities for further policy development. In particular, the development of contingency policies and guidelines for the safe continuation of recycling efforts in the context of pandemics stands to significantly contribute to unburdening the waste management chain down the line. In California, for instance, guidelines for recycling and composting operations were adapted to ensure service continuity despite the pandemic, with the addition of a newly enacted mitigation measure of waiting for at least 3 days before recyclable wastes are physically sorted, so as to ensure the safety of waste management personnel (79).

Moreover, there is also a need for policies to ensure the strengthening and adaptation of waste collection efforts during the pandemic and other public health emergencies. Studies have shown that municipal waste collection systems improve in efficiency and costs when collection bins are reallocated, collection vehicles are optimized for the traffic congestion in the area, and collection routes are optimized to maximize delivery time and decrease collection distance (80–82). In Vietnam, adaptive policies mandated the sealing of waste bags for collection, increasing collection frequency during the pandemic (at least twice a day), and treatment of collected waste within a day in compliance to several technical standards. Robust and strong enforcement of these policies have been documented to result in a lower number of COVID-19 transmission among waste handlers, with no deaths thus far (82).

Recognizing that waste management chains in the Philippines are not only vulnerable to the impacts of the COVID-19 pandemic but also to other natural hazards and disasters that frequent the country, we also recommend releasing provisional guidelines that will ensure the continuation of waste management efforts during the latter circumstances. Specifically, contingency guidelines must be established for the safe continuation of waste collection during emergencies and disasters, and the adaptation of waste storage, treatment, and end disposal guidelines to account for climate impacts (e.g., warming temperatures and increased precipitation).

### On strengthening accountability and transparency of waste management financing

Concrete policy directive for the strategic and responsive allocation of financial resources were found to be lacking in many of the policies reviewed. In the absence of a comprehensive financing strategy, the burden often falls disproportionately upon local governments to supply the resources needed for implementation–many of whom may not have these financial resources to begin with, particularly those in low-income and remote, rural locations. Contingency policies that provide for supplementary co-financing of waste management efforts by national government agencies, along with providing options for resource sharing between local governments and incentivizing public-private partnerships, may address the inherent constraints that delimit optimal financing and resource mobilization for waste management efforts during the pandemic.

Policy development may also be leveraged not only for increasing budgetary allocations and resource mobilization for waste management efforts but also in ensuring transparency and accountability–both of which are often set during public health emergencies such as the COVID-19 pandemic. By virtue of the 1987 Philippine constitution and the 1991 Local Government Code, government budgets are to be made publicly accessible, and the call for transparency in the public financial management of waste management efforts should be reinforced.

### On capacity-building and implementation

The key to avoid the escalation and crippling of the waste management system is prompt government response with strict protocols and regulations on the national and local level especially at the early stages of any potential waste management crisis. Government support should also be increased at the municipality-level in order to adequately implement the policies (e.g., increased number of designated public waste bins, provision of color-coded plastic waste bags, involvement of homeowners in community-based practices to promote proactive participation within their own space).

To address the criteria of human resources and organizational capacity building, training for waste treatment and disposal facilities should also be given more focus to augment these stages, using more eco-friendly technology. Partnerships with the private sector can also be incentivized and the informal sector of waste management can be included in future policies to help the safe continuation of resource recovery (e.g., reusing, recycling, and composting activities). Lastly, the adoption of new technologies that will enable the extended use of resources (e.g., PPE's) should be explored to solve the scarcity in supply.

## Conclusions

Developing countries like the Philippines continue to deal with weak regulatory governance structures and the absence of resources and infrastructures vital to effective policy grounding and implementation (83, 84). These situate many countries in ASEAN at a disadvantage in the global progression of waste generation brought by rapid urbanization, and the ongoing COVID-19 pandemic.

Content analysis of the fifty (50) policy documents vis a vis the Cradle-to-End-of-Life framework revealed adequate provisional coverage across all life-cycle stages for hazardous healthcare waste and general solid wastes from domestic settings. However, the emerging category of household healthcare waste was poorly covered as it was neither defined nor specifically governed in any of the policies analyzed. Evaluation of the internal validity of the policies demonstrated weak fulfillment of criteria on adequate financing, organizational capacity building, monitoring and evaluation, and encouragement of opportunities and public participation. Rapid, adaptive policy generation is necessary in times of crises like the COVID-19 pandemic but has also resulted in gaps and inconsistencies that must be revisited and adapted to ensure that waste management policies deliver their intended goals and contribute to larger public health and environmental outcomes.

However, even with strong policies and provisional support, limited infrastructure and lack of absorptive capacities to manage exponential increases in healthcare waste will lead to gaps in implementation, especially in far-flung, low-income localities where availability and access to implementation resources widely vary. Ultimately, disregard of social vulnerabilities and lack of alternatives for low-income regions will result in the brunt of the impacts shouldered by poor communities and the informal economy.

A dedicated policy implementation review is needed to evaluate how these policies are carried out across LGUs in different provinces. We recommend the study of waste management campaigns and available technology at different levels of office per region, province, city, and municipality in order to identify weaknesses in practice, explore opportunities to optimize the process, and give more support to those that need it. This would also allow the crafting of future policies that would not only meet international standards but also ensure that the guidelines are tailor-fit and flexible enough to LGUs' capacities and needs.

Relying on the status quo, policy mechanisms to address waste management amidst current and future pandemics will not be viable in the long run; hence, these are expected to adapt and evolve over time, utilizing available technology and innovations. Effective solid waste management needs a whole of government, whole of society approach as both institutions and communities are affected and involved.

## Data availability statement

The raw data supporting the conclusions of this article will be made available by the authors, without undue reservation.

## Author contributions

All authors listed have made equally substantial, direct, and intellectual contribution to the work and share first authorship. All authors approved the work for publication.

## Funding

This research was sponsored by the University Research Council grant of the Ateneo de Manila University (Control Number: COVID-URC18 2020).

## Conflict of interest

The authors declare that the research was conducted in the absence of any commercial or financial relationships that could be construed as a potential conflict of interest. The handling editor XS declared a past co-authorship with the authors GA and SV.

## Publisher's note

All claims expressed in this article are solely those of the authors and do not necessarily represent those of their affiliated organizations, or those of the publisher, the editors and the reviewers. Any product that may be evaluated in this article, or claim that may be made by its manufacturer, is not guaranteed or endorsed by the publisher.
